# Burr hole monoportal endoscopic resection of a large choroid plexus cyst causing obstructive hydrocephalus: diagnosis, management and literature review

**DOI:** 10.1007/s00381-025-07072-0

**Published:** 2025-12-04

**Authors:** Nida Kalyal, Anca-Mihaela Vasilica, Kantharuby Tambirajoo, Andrew King, Naomi Sibtain, Bassel Zebian

**Affiliations:** 1 Department of Neurosurgery, King’s College Hospital, London, UK; 2 UCL Medical School, University College London Medical School, London, UK; 3 Department of Neuropathology, King’s College Hospital, London, UK; 4 Department of Neuroradiology, King’s College Hospital, London, UK; 5 Institute of Psychiatry, Psychology and Neuroscience, King’s College London, London, UK

**Keywords:** Choroid plexus, Choroid plexus cyst, Neuroendoscopy, Obstructive hydrocephalus

## Abstract

**Introduction:**

Choroid plexus cysts, the most common neuroepithelial cystic lesions, are rarely symptomatic and often discovered incidentally.

**Materials and methods:**

We report our experience of managing a 22-year-old man who presented with a short history of severe, progressive headache secondary to hydrocephalus as a result of obstruction at the level of the foramina of Monro.

**Results:**

High resolution MRI sequences demonstrated a large cystic lesion filling and expanding the foramina of Monro and extending into the third ventricle. Endoscopic gross total resection of the cyst and a septum pellucidotomy were performed through a monoportal approach with resolution of the hydrocephalus and the headaches.

**Conclusion:**

Although symptomatic choroid plexus cysts are rare, they should be considered in the differential diagnosis of intraventricular lesions causing obstructive hydrocephalus and appropriate imaging performed. Endoscopic resection is a safe and definitive intervention, which allows complete excision via a minimally invasive approach.

## Introduction

Choroid plexus cysts are the most common neuroepithelial cystic lesions. They are usually incidental asymptomatic lesions found on imaging or identified post-mortem. They are most commonly located in the atria of both lateral ventricles. Symptomatic cysts are rare, with symptoms attributed to large size and/or location resulting in obstructive hydrocephalus. The operative approaches described in the literature are either fenestration or microscopic resection. We report a case where advances in endoscopic techniques have allowed complete resection using a minimally invasive approach.

## Case report

### History and examination

The patient, a 22-year-old man with no previous medical history, presented to the emergency department with a one-week history of progressive headaches. There were no associated features of raised intracranial pressure but due to the severity of the headaches, computed tomography (CT) and subsequently magnetic resonance imaging (MRI) scans of the brain were performed. Clinical examination was unremarkable, with no neurological deficits detected. Formal ophthalmological testing was not performed as we did not believe it would alter the management.

### Radiology

**Fig. 1:**
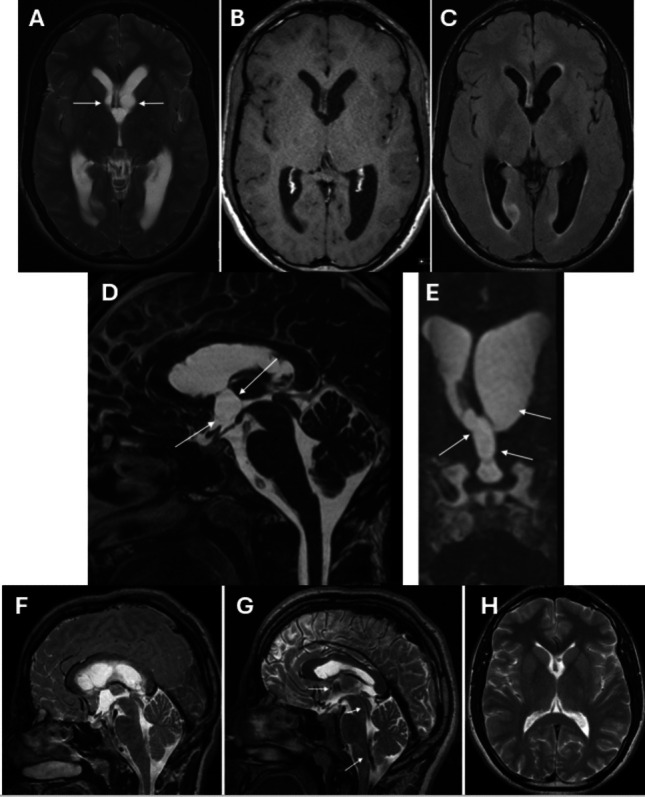
**A**, **B** and **C**—Pre-operative axial T2 (1A), T1 (1B) and FLAIR images (1C) demonstrate the cystic lesion involving both Foramina of Monro (arrows) and projecting into the third ventricle with ensuing hydrocephalus. Note that the lesion is isointense to CSF on all sequences. **D** and **E**—Mid-sagittal FIESTA image (1D) demonstrates the 3rd ventricular component of the cyst (arrows). Coronal reformat (1E) shows the cyst involving both foramina of Monro (larger on the left) and third ventricle (arrows). **F** and **G**—Mid-sagittal T2 SPACE image (1G) following cyst resection shows normalisation of CSF flow across the ventricular system with flow voids present across the foramina of Monro, aqueduct and fourth ventricular outlet foramina (arrows) that were absent or reduced on the corresponding pre-operative 2D multi-slice T2w FSE image (1F). **H—**Axial T2 image from 3.5 years follow-up MRI shows normal ventricular size with no recurrence of the cyst. (The tip of a ventricular catheter is visible in the left lateral ventricle)

Imaging demonstrated bilateral lateral ventricular enlargement with a non-enhancing CSF isointense lesion shown at the foramina of Monro and projecting substantially into the third ventricle (Fig. 1A, B and C).

### Operative procedure

**Fig. 2:**
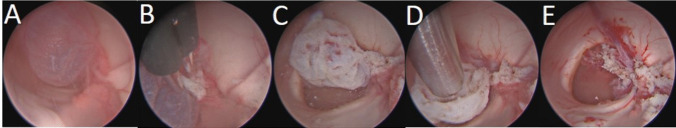
Endoscopic views illustrating (**A**) a cyst blocking the left foramen of Monro. It was coagulated (**B**) and shrunk (**C**) and removed in its entirety (**D**) revealing the foramen of Monro underneath (**E**)

The patient underwent an endoscopic resection of the cystic lesion through a left frontal burr hole approach under electromagnetic neuronavigation guidance (AxiEM, Medtronic Minneapolis, USA). On introducing the endoscope (Minop™InVent, Aesculap AG, Tuttlingen, Germany) (large working channel and 30-degree lens) into the left lateral ventricle, a large cyst obliterating the foramen of Monro on the left and extending into the third and right lateral ventricles was seen (Fig. 2). It was clear that the cyst was mobile with a pedicle arising from the choroid plexus in the region of the left foramen of Monro and causing intermittent obstruction through a ball-valve mechanism. This was demonstrated intraoperatively with the use of wash. Using bipolar diathermy, we coagulated the surface of the cyst to shrink it. We then used a combination of an endoscopic ultrasonic aspirator (Sonoca, Söring GmbH, Germany) which we modified to use down our specific endoscope and sharp dissection using endoscopic scissors to achieve a gross total resection with visualisation of the floor of the third ventricle. A septum pellucidotomy was also performed as the right foramen of Monro could not be visualised and inspected. A Rickham reservoir attached to a 6 cm ventricular catheter was inserted into the left lateral ventricle and left in situ to cover the bony defect and allow access should it become required.

### Histological analysis

**Fig. 3:**
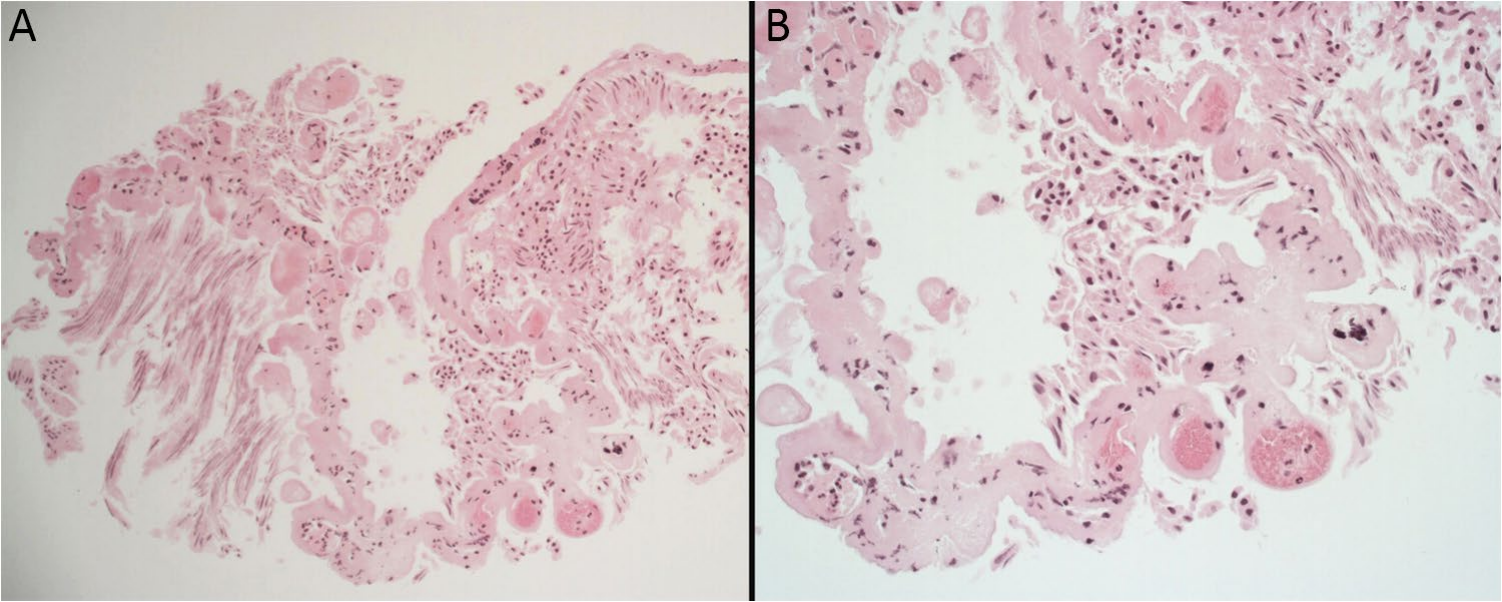
The histology at low power (**A**) and higher power (**B**) shows the wall of a cystic structure with an epithelial cell type lining arranged focally in a papillary pattern (**A**) and with an underlying fibrovascular core (**B**), being fully in keeping with a choroid plexus cyst. (H & E. Original magnifications A- × 10, B- × 20)

Microscopic sections of the lesion showed a cystic structure lined by partially crushed cuboidal-like epithelium arranged focally in a papillary pattern and with an underlying fibrovascular core (Fig. 3). Immunohistochemistry revealed strong immunopositivity for S100 and focal positivity for GFAP and EMA in the lining cells. These features were fully consistent with a choroid plexus cyst.

### Postoperative course

The patient made an excellent recovery with complete resolution of his headaches. Postoperative MRI showed resolution of hydrocephalus (Fig. 1H). Long-term follow-up over 6 years demonstrated no cyst recurrence and no longer-term morbidity related to the procedure or approach.

## Discussion

Choroid plexus cysts are often diagnosed on antenatal ultrasound scans. In the absence of other anomalies, they are largely incidental findings and either spontaneously regress by birth [1, 2] or continue as asymptomatic lesions into adulthood when they may be picked up as incidental findings on imaging or post-mortem, with some studies reporting that they are seen in up to 30% of autopsies [3]. They show no gender predilection, but their prevalence increases with age [4]. The vast majority are located in the atria of lateral ventricles bilaterally [5], where they are much less likely to obstruct CSF flow.

The imaging appearances of choroid plexus cysts are varied, reflecting the variable extent of lipid content, haemosiderin and peripheral psammomatous calcification. On CT, lesions may be mildly hyperdense or isodense to CSF and may show peripheral calcification. On MRI, the cysts are typically T1 isointense, T2 mildly hyperintense and FLAIR hyperintense to CSF [6]. Our case illustrates that if the lesion is isointense to CSF on all sequences, then a FIESTA (fast imaging employing steady state acquisition sequence) (or CISS sequence on a Siemens MR scanner) is invaluable in being able to demonstrate the thin cyst wall. Without this added information, the lesion may be overlooked, potentially resulting in underdiagnosis in patients presenting with persistent or intermittent symptoms of raised intracranial pressure and ventriculomegaly but with no clear lesion demonstrated.

Choroid plexus cysts causing obstructive hydrocephalus are rare, and thus, there is paucity in the literature regarding the management of such lesions. Management options for symptomatic lesions include a range of neurosurgical interventions, from shunting to fenestration and resection. Although shunting may relieve associated hydrocephalus with potentially less risk than the alternatives, it does not address the causative lesion, does not provide histological diagnosis and most importantly results in life-long shunt dependence with likely delayed additional surgery [7]. Cyst fenestration, while representing an alternative management option, is often associated with an increased risk of recurrence compared to surgical resection.

Craniotomy and microscopic fenestration or resection have been successfully performed and reported but with a greater need for supplementation by cysto-ventriculoperitoneal shunting when compared to endoscopy [8].

The endoscopic approach was previously thought to preclude complete resection of these lesions but, with improved instrumentation and technique, it is increasingly apparent that complete endoscopic resection is indeed achievable. Of 12 case reports of symptomatic choroid plexus cysts managed endoscopically, 6 had complete resection [7, 9–12] (Table 1). The more conservative intervention of fenestration [7], although relieves the obstruction, does not prevent recurrence of hydrocephalus caused by obstruction due to cyst remnants. Where cysts are fenestrated, long term follow up is required [13]. At present, longer term (5–10 years) follow-up studies to effectively compare recurrence rates of the different interventions are lacking.

**Table 1:** Endoscopic management of choroid plexus cysts

Reference	Age/Gender	Cyst Location/Level of Obstruction	Surgery	Follow up	Outcome
Parízek et al. [7]	16yo/M	Left Lateral Ventricle	CT Stereo-endoscopic resection	3 months	Good, no recurrence
Jeon et al. [10]	26yo/M	Trigone of Right Lateral Ventricle	Endoscopic resection	N/A	No recurrence; post-operative intraventricular haemorrhage, catheter infection
Miyagi et al. [12]	1yo/F	Third Ventricle	Endoscopic resection	18 months	Good, no recurrence
Nahed et al. [13]	2yo/M	Left Lateral Ventricle	Endoscopic fenestration	N/A	N/A
van Baalen et al. [14]	4mo/M	Third Ventricle	Endoscopic fenestration & ETV	N/A	N/A
Kariyattil et al. [11]	3yo/F	Third Ventricle	Endoscopic resection	3 months	Good, no recurrence
Freppel et al. [15]	6do/M	Left Lateral Ventricle	Endoscopic fenestration	18 months	Good, no recurrence
Filardi et al. [9]	11wo/F	Third Ventricle	Endoscopic resection	13 months	Good, no recurrence
Chamczuk et al. [16]	47yo/F	Left Lateral Ventricle	Endoscopic cauterisation	3 years	Good, no recurrence
Eboli et al. [17]	11wo/F	Third Ventricle	Endoscopic fenestration & ETV	24 months	Good, no recurrence
de Lara et al. [18]	25yo/F	Third Ventricle	Endoscopic cauterisation & ETV	N/A	Good, no recurrence
Azab et al. [19]	9yo/M	Third Ventricle	Endoscopic ablation & ETV	N/A	Good, N/A
Present study	22yo/M	Lateral Ventricles	Endoscopic resection & septum pellucidotomy	6 years	Good, no recurrence

*Do* Days-old; *ETV*  Endoscopic thir ventriculostomy; *F *Female; *M*  Male; *mo*  Months-olds; *NA* Not reported in the study; *wo* Weeks-old; *yo* Years-old

We opted for a gross total resection of the cyst to reduce the risk of recurrence. We also delayed publication to ensure longer-term follow-up of over 5 years is reported. Although our patient was a young adult at the time of surgery, the endoscopic principles described are applicable across paediatric and transitional age groups, as symptomatic choroid plexus cysts, although rare, can occur at any age. Furthermore, in our centre, teenage and young adult patients up to and including the age of 24 with pathology best addressed using endoscopic intraventricular approaches are managed by those with paediatric expertise.

## Conclusion

Acute obstructive hydrocephalus as a result of a choroid plexus cyst is rare. Nonetheless, it should be considered in the differential diagnosis of a patient presenting with raised intracranial pressure and ventriculomegaly affecting one or both lateral ventricles with or without involvement of the third ventricle. A FIESTA or CISS sequence may be required make the diagnosis by delineating the thin wall of the cyst. We demonstrated that monoportal endoscopic gross total resection through a burr hole is feasible and safe with no long-term recurrence in our patient. We recommend this approach as the first line management in centres with the appropriate expertise.

## Data Availability

No datasets were generated or analysed during the current study.
